# Center Hemodialysis Versus Peritoneal Dialysis: A Cost-Utility Analysis

**DOI:** 10.7759/cureus.55667

**Published:** 2024-03-06

**Authors:** Ludwig Matrisch, Yannick Rau

**Affiliations:** 1 Medical Clinic I, University Hospital Schleswig-Holstein, Lübeck, DEU; 2 General Practice, General Practice Teetzmann, Mölln, DEU

**Keywords:** health-related quality of life, kidney replacement therapy, chronic kidney disease, center hemodialysis, hemodialysis, peritoneal dialysis, dialysis, cost-benefit analysis, cost-utility analysis, quality of life

## Abstract

Introduction

Kidney replacement therapy (KRT) is needed for patients with end-stage kidney disease. While it is clear that kidney transplantation remains the gold standard in KRT, data comparing the cost-utility of peritoneal dialysis (PD) and hemodialysis (HD) are scarce. No such analysis has been performed for German patients.

Methods

We used aggregated data generated by the Short Form 36 Health Survey (SF-36) for quality of life and insurance claims to evaluate mortality and economic impact. Quality-adjusted life years (QALY) and cost-utility were calculated accordingly.

Results

PD is superior to HD within all dimensions of the SF-36, both in terms of QALY and cost-utility. The difference in cost per QALY between the aggregated physical dimensions (€50,671.54 vs. €39,745.77) is greater than that of the aggregated mental dimensions (€31,638.75 vs. €25,287.63). However, there is considerable variability among patients.

Conclusion

From a health-economic point of view, PD should be preferred over HD when deciding on the KRT modality for the patient. This is not reflected in current practice, though. However, interindividual differences and patient preferences should be considered in the decision.

## Introduction

End-stage kidney disease (ESKD) describes the last stage of chronic kidney disease (CKD). It is characterized by an aggravation of the associated outcomes known in CKD, such as anemia, mineral bone disease, and increased cardiovascular morbidity. Due to the high disease burden for the patient, kidney replacement therapy (KRT) is needed unless contrary preferences are stated by the patient. The modality of KRT with the best outcome is kidney transplantation [1]. Unfortunately, some patients are ineligible for kidney transplantation, mostly due to cardiovascular reasons [2]. Even for those who are eligible, the procedure is not always an option due to the scarcity of available transplant kidneys. Therefore, dialysis as KRT is highly prevalent.

There are two main types of dialysis: hemodialysis (HD) and peritoneal dialysis (PD). Both modalities provide kidney replacement through the diffusion of molecules in solution across a semipermeable membrane along an electrochemical concentration gradient. Additional conventional transportation can be added through the application of osmotic or hydrostatic pressure. The semipermeable membrane, however, differs between the modalities. While it is provided by an extracorporeal filter in HD, healthcare providers use the endothelium of the peritoneal capillaries in PD [3]. These two modalities of KRT come with great differences in terms of applicability for patients. While HD mostly takes place in specialized dialysis wards or dialysis centers, PD can be performed in various settings. The entirety of the differences between the methods exceeds the scope of this article and has been far better described elsewhere; however, it seems plausible that such differences imply consequences for hard endpoints, such as death. However, decisions involving medical treatments are not purely technical. Instead, they should include patients’ values and preferences. To quantify those, various patient-reported outcomes (PRO) have been established. Both quality of life (QoL) and health-related QoL (HRQoL) are such PROs [4]. These tools enable medical and political decision makers to include patients’ perspectives in their decision process. While patients’ values and preferences can still be best discussed in conversation, aggregated data are needed to guide policymakers to improve the healthcare system toward patient-centered care.

PROs such as QoL, together with the life expectancy, can be calculated into quality-adjusted life years (QALY). QALY are a commonly used outcome parameter in health economics. They can be used to compare the effect of therapeutic interventions or preventive measures, even across diverse patient collectives. By adding the cost to the equation, the calculation can be extended to a cost-utility analysis. This lays the foundation for an objective decision on the allocation of resources in healthcare.

The decision on the best forms of dialysis is one of the most important decisions in nephrology. Therefore, criteria upon which to base one’s decision are urgently needed. Although both HD and PD have been around for decades, cost-utility analyses comparing them are not very prevalent. Also, the country-specific properties of the healthcare systems around the world warrant an analysis that considers the particular situation in the country of interest. Despite its active nephrology community and high prevalence of ESKD, such an analysis has not yet been conducted in Germany. We try to fill this gap by providing such an analysis in this paper.

## Materials and methods

Data collection

Data were gathered from various data sources. Data on HRQoL were extracted from a meta-analysis by Chuasuwan et al. [5]. The authors retrospectively calculated HRQoL outcomes from 17 studies, including 26,956 patients. Data were collected using the Short Form 36 Health Survey (SF-36).

The SF-36 is a commonly used, validated questionnaire that measures patient-reported health [6]. The questionnaire has been in use since 1988. It consists of eight sections that represent the following eight dimensions of health: vitality (four questions), physical functioning (10 questions), bodily pain (two questions), general health perceptions (five questions), physical role functioning (four questions), emotional role functioning (three questions), social role functioning (two questions), and mental health or emotional wellbeing (five questions). Patients can state their answers on a 3-point Likert scale. The weighted answers are calculated into a score between 0 and 100 for each scale [6]. From those scales, two additional scales aggregating the mental and physical domains can be calculated. Often, a total/overall SF-36 is reported. However, according to the developers of the questionnaire, a total score cannot be calculated from it [7].

Data regarding mortality and healthcare costs, including transportation, were extracted from a paper by Shukri et al. [8]. They analyzed claims data from two large German health insurance companies. In all, 28,533 patients were included in the primary analysis. Exclusion criteria included kidney transplantation within the analyzed time period and a switch from HD to PD (or vice versa). To account for baseline differences within the PD and HD population, patients were matched using propensity scores that were established by performing a logistic regression with enrolment into the PD group (yes = 1/no = 0) as the outcome variable of the matching process. The covariates included were as follows: age, sex, region of residence, and health status at baseline measured as Charlson Comorbidity Index score [9]. This resulted in 436 matched pairs (each with one PD patient and one HD patient).

Survival rates were calculated using Kaplan-Meier estimates. The follow-up period was three years. The calculated healthcare costs included costs associated with hospital treatment, costs of treatment in ambulatory care, drug expenses, costs of medical and therapeutic aids, costs due to sick pay, and patient transportation costs and rehabilitation costs.

Data analysis

Data analysis was performed using Microsoft Excel (version 2207, Microsoft, Redmond, USA). For this analysis, Gaussian distribution in the primary data was assumed. QALY were calculated by multiplying the years of survival by the QoL calculated by the SF-36. The QALY were stratified by the subdomains of the SF-36. Cost-utility was calculated by dividing the healthcare cost per year by the QALY. A two-sided t-test was performed to test the differences between HD and PD. α was set at 0.05.

## Results

QALY

Seventeen studies, including 26,956 patients, were included in the analysis [5]. In all, 5610 patients were treated with PD and 21346 with HD. Most patients were from developed countries. The majority of studies included patients from Europe. The patient collective is similar to the German collective [8].

The results of the analysis of QALY are displayed in Table 1. PD is superior in terms of QALY within all dimensions of the SF-36. The greatest absolute difference is seen in the dimension of emotional role functioning (0.162 QALY, p < 0.001), which is also reflected in the difference between PD and HD in the aggregated mental domain (0.106 QALY, p < 0.001). In contrast, the smallest absolute difference can be observed in the physical role functioning domain (0.034 QALY, p < 0.001). In general, the absolute difference in QoL related to its physical dimensions between PD and HD is smaller than the difference in its mental dimensions. This is also reflected in a smaller absolute difference in QALY for the aggregated physical dimensions compared to the aggregated mental ones (0.088 vs. 0.106). However, this can be attributed to lower aggregated physical than aggregated mental QALY for both HD (1.076 and 1.723, respectively) and PD (1.163 and 1.828, respectively).

**Table 1:** Quality-adjusted life years HD vs. PD The table displays the comparison of QALY between HD and PD. The results of a two-sided t-test are stated as well. QALY, quality-adjusted life years; HD, hemodialysis; PD, peritoneal dialysis; HD-PD, difference between hemodialysis and peritoneal dialysis; SF-36, the Short Form 36 Health Survey

SF-36 category	QALY HD	Standard deviation HD	QALY PD	Standard deviation PD	QALY HD-PD	Standard deviation HD-PD	t-statistic	p-value
Vitality	1.349	0.619	1.484	0.582	-0.136	0.85	-14.715	<0.001
Physical rolefunctioning	1.015	0.842	1.048	0.829	-0.034	1.182	-2.620	<0.001
Bodily pain	1.641	0.599	1.776	0.581	-0.135	0.835	-15.115	0.009
General health	1.063	0.455	1.171	0.458	-0.108	0.646	-15.799	<0.001
Energy	1.237	0.472	1.299	0.472	-0.062	0.668	-8.755	<0.001
Social role functioning	1.6	0.592	1.671	0.591	-0.071	0.836	-7.997	<0.001
Emotional role functioning	1.508	0.877	1.67	0.851	-0.162	1.222	-12.387	<0.001
Emotional well-being	1.690	0.464	1.788	0.434	-0.098	0.635	-14.264	<0.001
Physical aggregated	1.076	0.6	1.163	0.587	-0.088	0.839	-9.707	<0.001
Mental aggregated	1.723	0.679	1.828	0.663	-0.106	0.949	-10.357	<0.001

Cost-utility

The analysis of cost-utility, measured in € per QALY, is displayed in Table 2. PD is more cost-effective than HD across all dimensions of the SF-36. The greatest absolute difference in cost-effectiveness is seen in the dimension of emotional role functioning (€8,461.39 per QALY, p < 0.001). In contrast, the smallest absolute difference can be observed in the emotional well-being domain (€6,384.441 per QALY, p < 0.001).

In general, the absolute difference in cost-effectiveness related to physical dimensions between PD and HD is smaller than the difference in its mental dimensions. This also reflects a smaller absolute difference in cost-effectiveness for the aggregated physical dimensions compared to the aggregated mental ones (€10,925.77 vs. €6,351.114). However, this can be attributed to a higher cost per QALY for the aggregated physical than aggregated mental dimensions for both HD (€50,671.54 and €31,638.75, respectively) and PD (€39,745.77 and €25,287.63, respectively).

**Table 2:** Cost-utility analysis HD vs PD The table displays the differences in € per QALY between HD and PD. The results of a two-sided t-test are provided as well. QALY, quality-adjusted life years; HD, hemodialysis; PD, peritoneal dialysis; HD-PD, difference between hemodialysis and peritoneal dialysis; SF-36, the Short Form 36 Health Survey

SF-36 category	€ per QALY HD	Standard deviation HD	€ per QALY PD	Standard deviation PD	€ per QALY HD-PD	Standard deviation HD-PD	t-statistic	p-value
Vitality	40,414.06	33,879.95	31,148.48	27,125.80	9,265.578	43,401.15	18.95	<0.001
Physical role functioning	53,708.90	45,965.09	44,104.85	38,447.26	9,604.05	59,924.80	14.383	<0.001
Bodily pain	33,202.74	32,854.59	26,028.51	27,179.89	7,174.235	42,640.01	15.057	<0.001
General health	51,287.69	24,923.94	39,494.62	21,342.50	11,793.07	32,813.18	32.451	<0.001
Energy	44,055.52	25,880.07	35,586.56	22,035.42	8,468.961	33,990.26	11.463	<0.001
Social role functioning	34,063.41	32,463.87	27,677.55	27,580.35	6,385.866	42,597.87	13.508	<0.001
Emotional role functioning	36,151.38	47,921.41	27,689.99	39,564.64	8,461.39	62,143.56	12.179	<0.001
Emotional well-being	32,244.81	25,568.83	25,860.37	20,452.93	6,384.441	32,742.74	17.304	<0.001
Physical aggregated	50,671.54	32,813.90	39,745.77	27,271.68	10,925.77	42,667.28	22.943	<0.001
Mental aggregated	31,638.75	37,199.70	25,287.63	30929.83	6,351.114	48,378.43	11.764	<0.001

## Discussion

To the best of our knowledge, this is the first cost-utility analysis comparing PD and HD for KRT in ESKD in Germany. This is an important topic that urgently needs reliable data in order to guide the public debate within the nephrology community, especially in Germany. Around 100,000 patients in Germany require KRT with dialysis [10]. This amounts to 0.12% of the whole population. This number is expected to grow by 20-23% until 2040 due to demographic changes in the population and a high incidence of risk factors for ESKD, such as diabetes and hypertension [10]. Thus, the health economics of KRT can be expected to become a topic of increasing interest in the upcoming years.

In other countries, similar analyses had already been conducted. Ferguson et al. found that, in Canada, the cost for one QALY in dialysis patients is $103,779, with PD being more cost-utile than HD ($83,762 and $104,880, respectively) [11]. They investigated the patients in a longer follow-up period, which might explain the discrepancies in our numbers. Researchers in Asian countries similarly calculated superior cost-utility of PD in their analyses [12]. The overall better cost-utility in their papers compared to ours or the one from Ferguson et al. might be attributable to a generally lower cost level and especially lower wage costs in the respective countries.

Given that the economic superiority of PD in comparison to HD has already been proven in various settings, it might come as a surprise that HD is much more prevalent in ESKD patients than PD [13]. In Germany, only about 6% use PD as their method of KRT [14]. The underlying reasons are manifold. Firstly, some patients are not eligible for PD due to medical reasons, such as morbid obesity, previous major abdominal surgeries, or large aneurysms of the aorta abdominalis. Such contraindications exist in 22% of patients with newly diagnosed ESKD [15]. Additionally, 63% of the patients without contraindications are hindered from receiving PD by barriers such as impaired vision, immobility, or psychiatric illness [15]. These barriers might lead nephrologists to nudge their patients toward HD in the decision process. They can often be overcome by effective education programs conducted by healthcare providers. However, patients are often referred to a nephrologist only very late in their CKD stage, leaving limited time for the educational process. Late referral has been shown to be an independent risk factor for the initiation of HD instead of PD [16]. Despite a legal obligation to inform patients about possible treatment options, only 56% of HD patients report being informed about other dialysis options by their healthcare provider [17]. Senior age and lower educational status were associated with lower probabilities of receiving such information [17]. Economic reasons might also play a role here. Although nephrologists denied financial factors leading their decisions in several survey studies, it has been shown that higher reimbursement of HD in comparison to PD leads to a higher share of ESKD patients receiving PD [18]. A low prevalence of PD could lead to a vicious circle of underutilization, as nephrologists and dialysis nurses have a hard time receiving proper training on PD. This might lead to the recommendation of HD for their patients as they feel more secure in offering the best method they are trained in.

In a global comparison, the prevalence of PD varies. While it is completely unavailable in some countries, it is still the most prevalent KRT modality in Hong Kong, being used by 73% of their patients [19]. Thus, clearly, there is potential for improvement for Germany. The high prevalence of PD is the result of a “PD first,” a campaign started in 1985 that centered around a reimbursement policy favoring PD [20].

Given that the cost per QALY is an abstract number, context is needed for interpretation. To estimate whether an intervention in healthcare can be considered cost-effective, one has to compare the cost-utility to other interventions. This can either be done, as demonstrated in this article, by comparing two interventions for the same condition or interventions for different conditions. The latter is important to decide whether public funding should instead go toward dialysis care or, e.g., vaccinations for children. In a broader context, policymakers have to decide on the proportion of the public budget that should go toward health interventions as opposed to, e.g., investments toward education. Some countries have adopted an explicit or implicit threshold for cost-utility for that question. In the USA, $50,000 per QALY has long served as a benchmark for cost-utility. However, recent analyses tended to increase that number to $100,000 or even up to $150,000 [21]. Countries with lower economic performance might set lower thresholds. Either way, judging by the results of this analysis, both HD and PD can be considered cost-effective in the context of Germany.

In a changing field like medicine, it is important to anticipate future developments. Recently, the focus of nephrology has shifted toward the ecological impact of dialysis. Due to climate change, reducing the greenhouse gas (GHG) footprint has become the target for any industry. Data comparing the GHG emissions between HD and PD are still scarce; however, there is evidence suggesting that these emissions might be lower in PD [22]. With the increasing importance of the climate impact on healthcare, this might nudge healthcare providers to favor PD over HD. However, the current paradigm on optimal dialysis might change in the future, as recent data have shown hemodiafiltration to be superior to HD in terms of mortality [23]. How this novel knowledge will be implemented in the future and how hemodiafiltration holds up in terms of medical outcome and cost-utility are still unknown, and future research is needed to improve patient care in this area.

Limitations

Although the results presented here are based on primary data acquired with sophisticated methodology, the analysis has its limitations. Firstly, this is a secondary analysis combining data sets that have been collected on different patient collectives. The QoL data used here is based on a broad foundation of global data; however, it might not be exactly representative of Germany due to differences in culture, patient attitudes, economic power, and the healthcare system between the countries. Secondly, the follow-up period of the mortality and healthcare cost analysis was only three years. While some patients can end dialysis due to receiving a transplanted kidney after three years, this is not the case for every patient. Thirdly, due to the usage of aggregated data, correlations between the examined parameters cannot be taken into account. Fourthly, there is considerable interpatient variability in the results. While PD is more cost-utile on average, the results could vary from patient to patient. Due to the use of aggregated data, the variability might be overestimated in our analysis, though. An individualized approach for the optimal KRT modality is warranted, and this should be reflected in healthcare policy.

## Conclusions

Due to the high prevalence and disease burden of ESKD, the decision on the optimal dialysis modality is of eminent importance for healthcare providers and for healthcare policy decision makers. In this paper, we have provided a cost-utility analysis comparing HD to PD using QALY as the outcome parameter. Across all dimensions of QoL, PD was superior to HD in terms of QALY and cost-utility. Still, only a few patients use PD, most likely due to medical barriers/contraindications and structural reasons. This should be considered for future healthcare policy decisions. However, considerable interindividual variability could be observed. Thus, interindividual differences and patient preferences should be considered when making a decision for either KRT modality.
